# WHAT I KNOW NOW THAT I WISH I HAD KNOWN WHEN I WAS YOUNGER: OLDER WOMEN’S RELATIONSHIP ADVICE AS SKINNY POETRY

**DOI:** 10.1093/geroni/igac059.3003

**Published:** 2022-12-20

**Authors:** Wendy Watson, Sandra Faulkner, Charlie Stelle

**Affiliations:** Bowling Green State University, Bowling Green, Ohio, United States; Bowling Green State University, Bowling Green, Ohio, United States; Bowling Green State University, Bowling Green, Ohio, United States

## Abstract

Research has demonstrated that relationships are important in the lives of older women. Eighteen women between the ages of 64–84 participated in interviews about their lives and relationships across the life course. As a final question, the women were asked, “What do you know now about relationships that you wish you’d known when you were young?” Responses to that question were examined using a skinny poem. The Skinny poem is a short poetry form that portrays a vivid image in 11 “skinny” lines. It is a type of lyric poem that uses condensed images, expressions of personal feelings, and demonstrations of emotional context in musical lines. Skinnys were written by one author and analyzed by a second author. This poster will present the 18 skinny poems and the analysis of the poems. In these skinnys, one can see the relationship experiences the women had throughout their lives. There is a connection to wisdom, a recognition that they have grown and learned about relationships over time and have advice they wished their younger selves had known. Emotions range from anger and anxiety to trepidation and regret. Most of the women reflect on things they would have done differently, from how they treated others to how they felt about themselves. They recognize that relationships take work and that as older women, they still do not know everything about relationships. They also learned about themselves as individuals and wished they had not searched for their identity and self-worth in their connection to others.

